# Acute kidney injury secondary to thrombotic microangiopathy associated with idiopathic hypereosinophilic syndrome: a case report and review of the literature

**DOI:** 10.1186/s13256-019-2187-4

**Published:** 2019-09-05

**Authors:** Diana Curras-Martin, Swapnil Patel, Huzaif Qaisar, Sushil K. Mehandru, Avais Masud, Mohammad A. Hossain, Gurpreet S. Lamba, Harry Dounis, Michael Levitt, Arif Asif

**Affiliations:** Department of Medicine, Internal Medicine Residency Program, Jersey Shore University Medical Center, Hackensack Meridian Health, Neptune, NJ 07753 USA

**Keywords:** Idiopathic hypereosinophilic syndrome, Eosinophilic cytotoxicity, Thrombotic microangiopathy

## Abstract

**Background:**

Renal involvement in idiopathic hypereosinophilic syndrome is uncommon. The mechanism of kidney damage can be explained as occurring via two distinct pathways: (1) thromboembolic ischemic changes secondary to endocardial disruption mediated by eosinophilic cytotoxicity to the myocardium and (2) direct eosinophilic cytotoxic effect to the kidney.

**Case presentation:**

We present a case of a 63-year-old Caucasian man who presented to our hospital with 2 weeks of progressively generalized weakness. He was diagnosed with idiopathic hypereosinophilic syndrome with multiorgan involvement and acute kidney injury with biopsy-proven thrombotic microangiopathy. Full remission was achieved after 8 weeks of corticosteroid therapy.

**Conclusion:**

Further studies are needed to investigate if age and absence of frank thrombocytopenia can serve as a prognostic feature of idiopathic hypereosinophilic syndrome, as seen in this case.

## Introduction

Idiopathic hypereosinophilic syndrome (HES) is characterized by an absolute eosinophil count greater than 1500 cells/mm^3^ observed at least twice with a minimum interval of 4 weeks, multiorgan involvement, and presence of tissue damage without an identifiable underlying cause [1]. Renal involvement in HES varies from 7% to 36%; however, kidney injury mediated by thrombotic microangiopathy (TMA) is rare [2]. To the best of our knowledge, only two cases of idiopathic HES [3] and one case of myeloproliferative-variant HES [4] have been reported. None of the reported cases achieved normal kidney function after treatment. The pathophysiology of renal impairment in HES can be explained by two mechanisms: (1) an ischemic kidney injury secondary to cardiac mural thrombus mediated by eosinophilic cytotoxicity to the heart (endocardium and myocardium) and (2) direct eosinophilic cytotoxic effect to the kidney [2–5]. We present a rare case of idiopathic HES with multiorgan failure and renal biopsy-proven TMA in which complete remission was achieved after 8 weeks of steroid therapy.

## Case presentation

A 63-year-old Caucasian man presented to our hospital with 2 weeks of progressive generalized weakness, vague abdominal discomfort, and dyspnea on exertion requiring more frequent use of his inhaler. He did not report similar symptoms in the past, and he denied any associated chest pain, cough, changes in bowel habits, fevers, chills, weight loss, recent travel, tick bites, or sick contacts. His past medical history was relevant for chronic bronchitis diagnosed 10 years ago. He was a former one-pack-per-day smoker for 20 years. His family history was noncontributory.

### Clinical findings

The patient’s vital signs at presentation showed a blood pressure of 128/84 mmHg, heart rate of 75 beats/minute, respiratory rate of 18 breaths/minute, oxygen saturation of 99% on room air, and body temperature of 97.7 °F. On physical examination, the patient was in no apparent distress and was awake, alert, and oriented to person, place, and time. His heart and lung examination revealed sinus tachycardia and diffuse expiratory wheezes throughout the lung fields. The patient’s abdominal examination was pertinent for a nonperitonitic tenderness to palpation in the left upper quadrant. His neurological examination was remarkable for weakness in the right upper extremity. His laboratory data are summarized in Table 1.

**Table 1:** Laboratory data during hospitalization

Biochemistry	Results	Reference values
Sodium	133	136–145 mmol/L
Potassium	4.0	3.5–5.2 mmol/L
Chloride	101	96–110 mmol/L
Bicarbonate	22	24–31 mmol/L
Blood urea nitrogen	17	5–25 mg/dl
Creatinine	1.3	0.61–1.24 mg/dl
Glomerular filtration rate	56	> 60 ml/minute
Glucose	87	70–99 mg/dl
Protein, total	6.9	6.0–8.5 g/dl
Albumin	2.8	3.5–4.7 g/dl
Aspartate aminotransferase	40	10–42 IU/L
Alanine transaminase	51	10–60 IU/L
Alkaline phosphatase	128	38–126 IU/L
Total bilirubin	2.0	0.2–1.3 mg/dl
Lactate dehydrogenase	343	91–200 IU/L
Haptoglobin	41	40–268 mg/dl
Troponin I	0.13	> 0.08 ng/ml
Procalcitonin	0.11	< 0.05 ng/ml; low-risk sepsis
Complete blood count		
Hemoglobin	10.0	12.0–17.5 g/dl
Hematocrit	30.2	36–53
Red cell distribution width	17.0	11.5–15.0%
Mean corpuscular hemoglobin	28.9	26.0–34.0 pg
Platelets	190	140–450 × 10^3^/μl
White cell count	22.2	4.5–11.0 × 10^3^/μl
Neutrophil count	4.8	1.50–7.50 × 10^3^/μl
Eosinophil count	16.2	0.00–0.50 × 10^3^/μl
Erythrocyte sedimentation rate	32	0–15 mm/hour
International normalized ratio	1.15	0.88–1.15

### Timeline

**Fig. 1:**
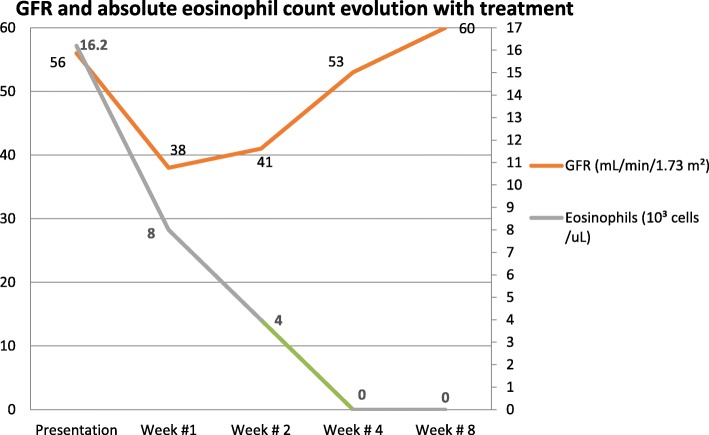
Kidney function and absolute eosinophil count evolution on steroid treatment. *GFR* Glomerular Filtration Rate

See Fig. 1 for the timeline of the patient’s kidney function and absolute eosinophil count while receiving steroid treatment.

### Diagnostic assessment

Findings of computed tomography (CT) of the patient’s brain were unremarkable. Magnetic resonance imaging (MRI) of his brain revealed subacute infarcts involving the left frontal white matter and left cerebellum; in addition, an evolving subacute infarct was seen in the left corona radiata. CT of the chest demonstrated diffuse ground-glass opacity, and CT of the abdomen was remarkable for a wedge-shaped area of low attenuation in the spleen consistent with splenic infarct. His transthoracic echocardiogram revealed a mural apical thrombus in the left ventricular (LV) apex with reduced ejection fraction (31–35%). Cardiac MRI performed 7 days after anticoagulation therapy was initiated showed a diffuse subendocardial scarring of the middle to apical LV segments and the right ventricular side of the septum. It also revealed evidence of edema of the middle anteroseptum and apical septum, consistent with endomyocardial fibrosis. However, no mural thrombus was visualized.

A presumptive diagnosis of HES was made on the basis of presenting symptoms, laboratory data, and imaging studies. Investigation for secondary causes, including immunological testing (Table 2), blood and urine cultures, ova and parasites, and infectious serology (Table 3), were unrevealing, and results of urine drug screening were negative. Bone marrow biopsy demonstrated a normocellular bone marrow population with eosinophilia comprising 60–70%, without evidence of lymphoproliferative disorder or metastatic neoplasm. Cytogenetic analysis was unrevealing: negative for breakpoint cluster region-Abelson murine leukemia viral oncogene homolog 1 (*BCR-ABL1*) fusion, eosinophilia-associated platelet-derived growth factor receptor alpha (*PDGFRA*), platelet-derived growth factor receptor beta (*PDGFRB*), fibroblast growth factor receptor 1 (*FGFR1*), Janus kinase 2 (*JAK2*) mutation, and JAK2 pericentriolar material 1 (*PCM1*) fusion.

**Table 2:** Immunological test results

Autoimmune serology	Results	Reference values
Antinuclear antibody	0.50	< 0.90, negative
Anti-double-stranded DNA antibody	1	< 4 IU/ml, negative
Serine protease 3 antibody	1	0–19 AU/ml
Antineutrophil antibody	Positive	Negative
Myeloperoxidase antibody	Not detected	< 1.0, not detected
Histone antibody IgG	0.6	0.0–0.9 units
Cardiolipin IgA	0.0	0–11 units
Cardiolipin IgM	7.0	< 20 units, negative
Complement		
C4	15.6 → 15.4	16–40 mg/dl
C3	91.7 → 77	85–170 mg/dl
Immunoglobulins		
IgG	1240	600–1560 mg/dl
IgM	61	400–300 mg/dl
IgA	104	70–450 mg/dl
IgE	259	< 214 Ku/L

*DNA* Deoxyribonucleic acid, *Ig G* Immunoglobulin G, *Ig M* Immunoglobulin M, *Ig A* Immunoglobulin A, *Ig E* Immunoglobulin E

**Table 3:** Infectious disease test results

Infectious serology	Results	Reference values
Human immunodeficiency virus	Negative	Negative
Hepatitis C virus	Negative	Negative
Hepatitis A virus IgM	Negative	Negative
Hepatitis D virus	Negative	Negative
Hepatitis B core IgM	Negative	Negative
Hepatitis B surface antibody	Negative	Negative
Hepatitis B surface antigen	Negative	Negative
QuantiFERON interpretation	Indeterminate	
QuantiFERON-TB	0.00	0.00 IU/ml
*Aspergillus* antigen	Not detected	
*Aspergillus fumigatus* IgG	Negative	Negative
*Micropolyspora faeni* IgG	Negative	Negative
Pigeon serum	Negative	Negative
*Thermoactinomyces* *candidus*	Negative	Negative
*Thymus vulgaris*	Negative	Negative
*Setaria viridis*	Negative	Negative
*Strongyloides* antibody	Negative	Negative

*IgG* Immunoglobulin G, *Ig M* Immunoglobulin M

Due to the stigma of hemolysis (normocytic acute anemia, elevated lactate dehydrogenase and bilirubin, positive schistocytes with relative thrombocytopenia), further investigation was pursued. The result of the Coombs test (direct and indirect) was negative. A disintegrin-like and metalloprotease with thrombospondin type 1 motif 13 (ADAMTS13) activity level was greater than 50%, and the expression of complement regulatory proteins CD59 and CD55 on erythrocytes was within normal limits as determined by flow cytometry.

Due to a further decline in the estimated glomerular filtration rate (GFR) early in the patient’s hospital course, a kidney biopsy was pursued. Renal biopsy revealed a glomerular and vascular TMA, interstitial fibrosis, and inflammation with focal eosinophils (Fig. 1). IHC staining for eosinophil granule major basic protein 1 (MBP1) was not performed.

### Therapeutic intervention

Our patient was started on prednisone 1 mg/kg daily and a heparin protocol at 18 U/kg/hour with an activated partial thromboplastin time goal of 60–100 seconds. Simultaneously, warfarin was initiated. Once the patient’s international normalized ratio was within therapeutic range (2.0–3.0), he was anticoagulated with heparin and warfarin for an additional 48 hours. His eosinophil count and estimated GFR were monitored on an outpatient basis, and his prednisone dose was gradually tapered. After the eighth week, the patient was maintained on 5 mg of prednisone daily.

### Follow-up and outcomes

By the time the renal biopsy report was available, the patient’s kidney function had started to recover; hence, no further intervention was required. After initiation of treatment with steroids, the patient achieved resolution of pulmonary, cardiac, neurologic, and abdominal symptoms. Repeat echocardiography after 5 weeks showed improvement of LV ejection fraction to 50–55%. Complete normalization of eosinophil count and renal function was observed after 4 and 8 weeks of therapy, respectively (Fig. 1). At his 10-week follow-up, the patient continued to do well under close surveillance for renal and cardiac complications. At 12-month follow-up, he continued to have a normal eosinophil count and renal function. However, cardiac MRI showed persistent endocardial fibrosis.

## Discussion

HES is an uncommon disorder, marked by overproduction of eosinophils, eosinophilia greater than 1500/mm^3^, tissue infiltration, and organ damage. Idiopathic HES requires exclusion of primary and secondary causes of hypereosinophilia as well as lymphocyte-variant hypereosinophilia [1]. For the period from 2001 to 2005, the National Cancer Institute Surveillance, Epidemiology, and End Results Program reported an age-adjusted incidence of 0.036 per 100,000 person-years for myeloproliferative HES. The incidence of idiopathic HES remains obscure [6]. The most common presenting symptoms are weakness, fatigue, cough, and dyspnea, followed by fever, rash, rhinitis, and in rare cases angioedema [7]. The mortality of HES is close to 10%, with the leading cause of death attributed to cardiac events followed by thromboembolic phenomena [8].

The pathogenesis of HES is mediated by “piecemeal degranulation” or eosinophil activation and secretion of the granule cationic proteins (such as eosinophil peroxidase, eosinophil cationic protein, eosinophil**-**derived neurotoxin, and MBP1) and eosinophil-expressed cytokines (such as RANTES [regulated on activation, normal T expressed and secreted] and interleukin [4, 9]). Eosinophil granule cationic proteins have the capability to activate inflammatory cells such as mast cells to induce inflammatory mediators and direct tissue-damaging cytotoxicity. These multiple proinflammatory activities lead to endothelial damage, thrombosis by activation of complement and coagulation cascade, and direct platelet stimulation and downregulation of thrombomodulin by MBP1 [5, 9].

Renal involvement in idiopathic HES is a rare entity, with only a handful of cases reported in the medical literature (Table 4) [10–21]. TMA is a life-threatening syndrome of systemic microvascular occlusions and is characterized by sudden or gradual onset of thrombocytopenia, microangiopathic hemolytic anemia, and renal or other end-organ damage [22]. It has been associated with diverse diseases and syndromes, such as systemic infections, cancer, pregnancy complications (for example, preeclampsia, eclampsia, HELLP [hemolysis, elevated liver enzymes, low platelet count] syndrome), autoimmune disorders (for example, systemic lupus erythematosus, systemic sclerosis, antiphospholipid syndrome), hematopoietic stem cell or organ transplant, severe hypertension, and cocaine-induced [22, 23]. Liapis *et al.* first reported two cases of TMA associated with idiopathic HES, along with a third case of myeloproliferative variant of HES in association with TMA [3] (Table 5). Of the cases described by Liapis *et al*., none had full renal recovery. In contrast, our patient did remarkably well with steroid and anticoagulation therapy. After discharge, his eosinophil count remained stable with resolution of renal injury with prednisone.

**Table 4:** Renal pathology in hypereosinophilia

Patient age and sex	Diagnosis	Renal pathology	Reference
14-year-old female	Idiopathic HES	Thromboembolism	Spry [10]
50-year-old male	Idiopathic HES	Necrotizing IgA nephropathy	Shah *et al.* [11]
40-year-old male	Idiopathic HES	Interstitial nephritis	Bulucu *et al.* [12]
67-year-old woman	Idiopathic HES	Crescentic glomerulonephritis	Richardson *et al.* [13]
18-year-old male	Eosinophilic gastroenteritis	Immunotactoid glomerulonephritis	Choi *et al.* [14]
55-year-old male	Idiopathic HES	Interstitial nephritis, focal segmental glomerulosclerosis	Motellon *et al.* [15]
73-year-old male	Idiopathic HES	Ischemic changes, interstitial nephritis	Navarro *et al.* [16]
42-year-old male	HES	Renal Infarct	Smith *et al.* [17]
59-year-old male	HES	Interstitial nephritis	Garella *et al.* [18]
80-year-old female	Idiopathic HES	TTP	Ohguchi *et al.* [19]
40-year-old male	Idiopathic HES	ATN, Charcot-Leyden crystalluria	Hirszel *et al.* [20]
52-year-old male	Idiopathic HES	Membranous glomerulonephritis	Frigui *et al.* [21]

*HES* Hypereosinophilic syndrome, *IgA* Immunoglobulin A, *ATN* Acute tubular necrosis, *TTP* Thrombotic thrombocytopenic purpura

**Table 5:** Reported thrombotic microangiopathy cases: comparative table

Demographics	Our patient	Historical 1 [3]	Historical 2 [3]	Historical 3 [4]
Sex	Male	Male	Male	Male
Age, years	63	15	26	24
Race	Caucasian	African American	Caucasian	Caucasian
CBC at admission				
Hemoglobin 12.0–17.5 g/dl	10.0	9.8	13.3	10.7
Platelets 140–450 × 10^3^/μl	235^a^	76	101	130
White cell count 4.5–11.0 × 10^3^/μl	22.2	23.7	14.7	23.5
Differential count, %				
Neutrophils 1.50–7.50 × 10^3^/μl	4.8	8.3	6.7	Not available
Lymphocytes 1.50–3.70 × 10^3^/μl	0.9	2.8	2.6	Not available
Monocytes 0.20–1.00 × 10^3^/μl	0.2	0.7	0.4	Not available
Eosinophils 0.00–0.50 × 10^3^/μl	16.2	11.1	4.9	18.3
Schistocytes	Positive	Not available	Positive	Positive
Renal function test at admission				
Creatinine 0.61–1.24 mg/dl	1.3	10.9	2.2	1.81
GFR	56	Not available	57	50
Percentage eosinophils in bone marrow	60%	80%	44%	37%
Renal biopsy	Glomerular capillary thrombosis Mesangiolysis Endotheliosis Eosinophilic infiltrate	Arterial thrombosis Mesangiolysis Eosinophilic infiltrate	Arteriolar thrombosis Glomerular capillary thrombosis Eosinophilic infiltrate	Arteriolar thrombosis Glomerular capillary thrombosis Eosinophilic infiltrate
Urinalysis				
Proteinuria	Negative	Present	Present	Present
Red blood cells/high-power field	20	50	Not available	100
Eosinophils/high-power field	None	None	7	None
Treatment	Prednisone	Prednisone Rituximab	Prednisone Imatinib	Imatinib
Follow-up	At 2 months	At 6 months	At 12 months	At 12 months
Creatinine 0.61–1.24 mg/dl	1.18	5.2	1.8	1.7

*Our patient’s platelets at baseline: 377–348 × 10^3^/μl

*CBC* Complete Blood Count, *GFR* Glomerular Filtration Rate

The mechanism of kidney damage in TMA with HES can occur via two different pathways: (1) direct eosinophilic cytotoxic effects to the renal vasculature and (2) ischemia secondary to thromboembolic events due to endocardial disruption. Subsequently, endothelial damage and complement cascade activation will result in TMA [1, 2, 5]. Similarly to our patient’s case and cases reported previously, Spry [10] reported that one of every five patients with HES developed hypertension and some degree of proteinuria. However, the described patients presented late in the course of HES and most likely had ischemic changes to the kidney secondary to cardioembolism rather than intrinsic eosinophilic cytotoxicity [2].

A multicenter analysis demonstrated that steroids alone induced partial or complete response at 4 weeks of treatment in 85% of the patients [24]. It was also observed that patients with positive factor interacting with PAPOLA [poly(A) polymerase alpha] and CPSF1 (cleavage and polyadenylation specificity factor) (*FIP1L1*)–*PDGFRA* gene fusion had a higher response to imatinib than those without [24]. It has been hypothesized that the deletion of genetic material as occurs in HES may result in gain of fusion proteins [25].

We report the only patient treated solely with a steroid, and a complete resolution of acute kidney injury was achieved, in contrast to previously reported cases. We hypothesize that factors such as the patient’s age group, proteinuria, and relative thrombocytopenia might be important to consider as prognostic factors.

## Conclusion

TMA of the kidney in association with idiopathic HES is rare. To the best of our knowledge, this is the first case report of HES with multiorgan involvement that was successfully treated with a corticosteroid alone. Further studies are needed to investigate if age, absence of frank thrombocytopenia, and proteinuria can serve as prognostic features, as seen in our patient’s case.
